# Long-term effects on sexual function and fertility after treatment of testicular cancer

**DOI:** 10.1038/sj.bjc.6690424

**Published:** 1999-05

**Authors:** J T Hartmann, C Albrecht, H-J Schmoll, M A Kuczyk, C Kollmannsberger, C Bokemeyer

**Affiliations:** Department of Hematology/Oncology/Immunology/Rheumatology, UKL-Medical Center II, Otfried-Mueller-Straβe 10, Tübingen, D-72076, Germany; Department of Hematology/Oncology, Martin-Luther-University, Halle, 06120; Department of Urology, Hanover University Medical School, Hanover, 30625, Germany

**Keywords:** testicular cancer, sexual functioning, fertility aspects, chemotherapy, RPLND, irradiation

## Abstract

This retrospective study evaluates the types and incidences of sexual disturbances and fertility distress in patients cured from testicular cancer and examines whether there is an effect resulting from different treatment modalities. A self-reported questionnaire was sent to 124 randomly selected patients who were treated at Hanover University Medical School between 1970 and 1993. Ninety-eight patients were included in the study, representing a response rate of 78%. All patients had been in complete remission (CR) for at least 24 months. The median age at diagnosis was 28 years (range 17–44). The median follow-up at the time of study was 12.0 years (range 2.8–25.6). Twenty patients (20%) had been treated for seminomatous and 78 patients (80%) for non-seminomatous germ cell tumours. Treatment included surveillance (7%), primary retroperitoneal lymph node dissection (RPLND) (13%), chemotherapy (CT) (33%), CT + secondary resection of residual retroperitoneal tumour mass (SRRTM) (43%) and infradiaphragmatic radiotherapy (4%). Patients receiving two treatment modalities (CT+SRRTM) reported more frequent an unfulfilled wish for children. Inability of ejaculation was clearly associated with RPLND and SRRTM. Subjective aspects of sexuality, like loss of sexual drive and reduced erectile potential, occurred only in a minority of patients after treatment. No abnormalities were observed concerning the course of pregnancies of partners. In conclusion, sexual dysfunction and infertility are common long-lasting sequelae in testicular cancer survivors affecting approximately 20% of patients. The relative risk for infertility appeared to be elevated for patients treated with the combination of CT+SRRTM. Twenty-one of 40 patients were able to fulfil their wish for children, and no congenital abnormalities were observed in these children. © 1999 Cancer Research Campaign


*British Journal of Cancer* (1999) **80**(5/6), 801–807
© 1999 Cancer Research Campaign
Article no. bjoc.1998.0424

### Long-term effects on sexual function and fertility after treatment of testicular cancer

**JT Hartmann[1], C Albrecht[1], H-J Schmoll[2], MA Kuczyk[3], C Kollmannsberger[1] and C Bokemeyer[1]**

[1]Department of Hematology/Oncology/Immunology/Rheumatology, UKL-Medical Center II, Eberhard-Karls-University, Otfried-Mueller-Straβe 10, D-72076 Tübingen, Germany; [2]Department of Hematology/Oncology, Martin-Luther-University, 06120 Halle, [3]Department of Urology, Hanover University Medical School, 30625 Hanover, Germany

**Summary** This retrospective study evaluates the types and incidences of sexual disturbances and fertility distress in patients cured from testicular cancer and examines whether there is an effect resulting from different treatment modalities. A self-reported questionnaire was sent to 124 randomly selected patients who were treated at Hanover University Medical School between 1970 and 1993. Ninety-eight patients were included in the study, representing a response rate of 78%. All patients had been in complete remission (CR) for at least 24 months. The median age at diagnosis was 28 years (range 17–44). The median follow-up at the time of study was 12.0 years (range 2.8–25.6). Twenty patients (20%) had been treated for seminomatous and 78 patients (80%) for non-seminomatous germ cell tumours. Treatment included surveillance (7%), primary retroperitoneal lymph node dissection (RPLND) (13%), chemotherapy (CT) (33%), CT + secondary resection of residual retroperitoneal tumour mass (SRRTM) (43%) and infradiaphragmatic radiotherapy (4%). Patients receiving two treatment modalities (CT+SRRTM) reported more frequent an unfulfilled wish for children. Inability of ejaculation was clearly associated with RPLND and SRRTM. Subjective aspects of sexuality, like loss of sexual drive and reduced erectile potential, occurred only in a minority of patients after treatment. No abnormalities were observed concerning the course of pregnancies of partners. In conclusion, sexual dysfunction and infertility are common long-lasting sequelae in testicular cancer survivors affecting approximately 20% of patients. The relative risk for infertility appeared to be elevated for patients treated with the combination of CT+SRRTM. Twenty-one of 40 patients were able to fulfil their wish for children, and no congenital abnormalities were observed in these children.

**Keywords:** testicular cancer; sexual functioning; fertility aspects; chemotherapy; RPLND; irradiation

Testicular cancer is a curable cancer which mostly affects men between 20 and 35 years of age. Major progress was achieved with the introduction of cisplatin into combination chemotherapy regimens, yielding cure rates of 70–85% in patients with metastatic disease (Einhorn, 1990; Bokemeyer, 1998). Since most patients with testicular cancer are of a young age, the impact of therapy on sexual function and fertility has become increasingly important. Testicular cancer patients may have a reduced spermatogenesis (‘hypospermia’) at diagnosis (Hendry et al, 1983; Berthelsen and Skakkebaek, 1984; Cassileth and Steinfeld, 1987; Moynihan, 1987; Nijman et al, 1987; Fossa et al, 1988; Sleijfer et al, 1995) and after orchidectomy (Lampe et al, 1997), and only between 22 and 63% of patients fulfil the definition of normospermia at diagnosis (Hendry et al, 1983; Fossa et al, 1985; Nijman et al, 1987; Lampe et al, 1997). Three disease-associated conditions, local structural abnormalities detected by biopsy of the contralateral testis, the presence of sperm antibodies and endocrine factors, may be responsible (Berthelsen and Skakkebaek, 1984; Guazzieri et al, 1985). Treatment-related factors have additionally been identified, for example decrease of testosterone levels after orchidectomy. However, no strong correlation was demonstrable between testosterone serum concentration and erection (Buena et al, 1993; van Basten et al, 1995). Leydig cell function as well as testosterone production can be affected by radio- and chemotherapy (von Eschenbach, 1980; Aass et al, 1991). Irradiation may cause small vessels disease and peripheral neuropathy resulting in erectile dysfunction (Fossa et al, 1980; von Eschenbach, 1980; Tomic et al, 1983; Goldstein et al, 1984; Fossa et al, 1986). The possible organic–biologic effects of chemotherapy itself are also complex, including temporary reduction of testosterone levels, hyperprolactinaemia, induction of vascular damage and peripheral neuropathy (El-Beheiry et al, 1988; Aass et al, 1991; van Basten et al, 1997*a*). Retroperitoneal lymph node dissection (RPLND) may cause permanent dry ejaculation due to the surgical interruption of retroperitoneal sympathetic nerves (Drasga et al, 1983). The different effects of treatment on sexual functioning make their evaluation a complex problem, particularly in patients treated with more than one therapeutic modality. Large differences regarding the frequencies of sexual dysfunctions and the influence of different treatments are reported in the literature (Rieker et al, 1985, 1989; Schover and Eschenbach, 1985; Schover et al, 1986; Gritz et al, 1989; Tinkler et al, 1992; Aass et al, 1993; Bloom et al, 1993; Arai et al, 1997; Jonker-Pool et al, 1997). The aim of the present descriptive investigation was to evaluate the incidence of long-term effects on sexuality and try to identify treatment-related differences.

*Received 4 June 1998*
*Revised 10 November 1998*
*Accepted 20 November 1998*

*Correspondence to:* C Bokemeyer

This work represents part of the thesis of Ch. Albrecht at Hanover University Medical School.This paper was presented in part at the 9th European Cancer Conference on Clinical Oncology (ECCO 9), Hamburg, 14–18 September 1997.

### PATIENTS AND METHODS

The medical records of testicular cancer patients treated at the Department of Hematology/Oncology of Hanover University Medical School between 1970 and 1993 were reviewed. A written self-report questionnaire concerning sexual function was sent to 124 patients who were randomly selected and who had had no evidence of disease for more than 2 years. Since no adequate validated questionnaire was available at the begining of study it was designed in corporation with the Department of Medical Psychology and the Department of Gynaecology at Hanover University Medical School. The questionnaire consisted of 41 issues related to fertility and sexual function before and after treatment, e.g. decrease in sexual response (libido, sexual drive, erection, orgasm), ejaculation function, changes in sexual activity and satisfaction, as well as items concerning child-bearing, course of pregnancy and state of health of offspring, and demographics. For the variable ‘intensity of sexual life’ (including the ‘intensity of orgasm’ (0 = no orgasm, 1 = pronounced decrease, 2 = slighty decreased, 3 = normal feeling) and the ‘frequency of sexual intercourse’ (0 = never, 1 = rarely, 2 = frequently, 3 = very often)) answers were ranked on a four-point scale (score). Medical information was obtained from the patients’ records. Clinical staging of patients was available according to the Lugano Classification (Cavalli et al, 1980). Patients were divided into four subgroups, according to the following treatment modalities: (1) orchidectomy and surveillance, SV; (2) primary retroperitoneal lymph node dissection, RPLND; (3) cisplatin-based combination chemotherapy, CT; (4) combination of chemotherapy and secondary surgery, CT+SRRTM. Four patients who had received infradiaphragmatic radiotherapy (RT) were not considered in the analysis comparing different treatment modalities due to the small number of patients available. The applied chemotherapy regimens are summarized in Table 1 (Williams et al, 1987; Loehrer et al, 1988; Einhorn, 1990; Harstrick et al, 1991; Schmoll et al, 1993; Bokemeyer et al, 1996). The differences in sexual dysfunctions between the treatment subgroups were tested with the $\chi^2$-test or Fisher’s exact test for categorial variables, and Kruskal–Wallis test and the Wilcoxon test for continuous variables. Differences were considered to be statistically significant at *P*-values less than 0.05.

### RESULTS

### Patients’ characteristics

Of 124 evaluable patients from our registry, 26 patients (21%) did not return the questionnaire. Thus, aspects of sexuality and fertility were assessed in 98 men at a median follow-up period of 12.0 years after the diagnosis of testicular cancer (range 2.8–25.6). Twenty patients (20%) had been treated for seminomatous and 78 (80%) for non-seminomatous germ cell tumours. Treatment included RPLND alone in 13 patients (13%), radiotherapy (RT) alone in four patients (4%), CT alone in 32 patients (33%), and 42 patients underwent CT+SRRTM (43%). Seven patients underwent orchidectomy alone and surveillance for stage I disease (7%). Patients’ characteristics are outlined in detail in Table 2.

### Pretreatment fertility status and fatherhood after treatment

Thirty-nine (40%) patients had fathered 53 children before the diagnosis of testicular cancer (Table 2). All 53 children with a median age of 14.5 years (range 12.2–17.3) at the time of this investigation were reported to have developed normally. No pregnancies occurred during any cancer treatment.

Twenty-one of 40 (53%) patients who reported a wish for children had fathered children at a median time of 54 months after the end of treatment (3–108 months). Additionally, two women interrupted gravidity at 8 months and at 2 years after the end of cancer treatment of their male partner. Nineteen of 40 (48%) patients who tried to obtain conception, have been unsuccessful after a median duration of nearly 5 years. Pathological semen analysis (‘azoospermia’/< $1 \times 10^6$ $ml^{-1}$) could be identified in 15 patients (79%) and permanent dry ejaculation in one patient (5%). Two patients were found to suffer from psychosocial distress causing reduced erectile potential (11%). In one case the spouse did not want to attain pregnancy (5%).

**Table 1** Type of chemotherapy (No. of patients = 74)

| | | | |
|---|---|---|---|
| PEB | cisplatin | (20 mg m–2; days 1–5) | 40 (67%) |
| | etoposide | (100 mg $m^{-2}$; days 1–5) | |
| | bleomycin | (30 mg; days 2, 9, 16) | |
| PVB | cisplatin | (20 mg $m^{-2}$; days 1–5) | 8 (11%) |
| | vinblastine | (0.2 mg $kg^{-1}$; days 1, 2) | |
| | bleomycin | (30 mg; days 2, 9, 16) | |
| PEI | cisplatin | (20 mg $m^{-2}$; days 1–5) | 5 (7%) |
| | etoposide | (75 mg $m^{-2}$; days 1–5) | |
| | ifosfamide | (1.2 g $m^{-2}$; days 1–5) | |
| PEBOI | cisplatin | (50 mg $m^{-2}$; days 1–3) | 8 (11%) |
| | etoposide | (170 mg $m^{-2}$; days 1–3) | |
| | bleomycin | (15 mg $m^{-3}$; days 1, 8, 15, 22) | |
| | vincristine | (2 mg; days 1, 8, 15, 22) | |
| | ifosfamide | (5 g $m^{-2}$; day 15) | |
| CEB | carboplatin | (400 mg $m^{-3}$ (GFR-corrected); day 1) | 7 (10%) |
| | etoposide | (120 mg $m^{-2}$; days 1–3) | |
| | bleomycin | (30 mg; days 2, 9, 16) | |
| Carboplatin-mono | | (400 mg $m^{-2}$ (GFR-corrected); day 1) | 6 (8%) |

GFR = glomerular filtration rate.

*© Cancer Research Campaign 1999*

**Table 2** Characteristics of 98 testicular cancer patients according to treatment modality

| | | Treatment groups (No. of patients) | | | | |
|---|---|---|---|---|---|---|
| | **All (patients)** | **SV** | **RT** | **RPLND** | **CT** | **CT+SRRTU** |
| Eligible patients | 98 | 7 (7%) | 4 (4%) | 13 (13%) | 32 (33%) | 42 (43%) |
| Median current age (years) | 38.5 | 39 | 47 | 40 | 37 | 40 |
| Range | (25–55) | (30–52) | (37–53) | (30–50) | (27–53) | (25–55) |
| Months from diagnosis | 120 | 97 | 164 | 169 | 110 | 141.5 |
| Range | (34–307) | (55–259) | (109–230) | (34–238) | (73–171) | (43–307) |
| Median age at diagnosis (years) | 28 | 32 | 32 | 28 | 29 | 27 |
| Range | (17–44) | (23–44) | (29–40) | (19–43) | (19–44) | (17–41) |
| Histology | | | | | | |
| Seminoma | 20 (100%) | 2 (29%) | 4 (100%) | 3 (23%) | 5 (16%) | 6 (14%) |
| Non-seminoma | 78 (98%) | 5 (71%) | – | 10 (77%) | 27 (84%) | 36 (86%) |
| Stage (according to Lugano classification) | | | | | | |
| I | 24 (92%) | 7 (100%) | 4 (100%) | 9 (69%) | – | 4 (10%) |
| II | 50 (91%) | – | – | 3 (23%) | 18 (56%) | 29 (69%) |
| III | 17 (89%) | – | – | – | 10 (31%) | 7 (17%) |
| (Ne) | 7 (100%) | – | – | 1 (8%) | 4 (13%) | 2 (5%) |

RPLND, retroperitoneal lymph node dissection; CT+SRRTU, chemotherapy and retroperitoneal lymph node resection (mostly lumpectomy); CT, chemotherapy; RT, radiotherapy; SV, surveillance; NE, not evaluable.

In relation to the treatment modalities, patients treated with CT+SRRTM reported an unfulfilled wish for children more often (31%) as compared to patients belonging to the CT (13%, $P$ = 0.03), SV (14%), or RPLND (7%) groups. Twenty of 87 patients (20%) scheduled to receive CT or RPLND were offered sperm cryopreservation before treatment. Only eight of those patients (40%) had accepted sperm-banking, two of whom were planning in-vitro fertilization (25%). None of the infertile patients planned to adopt children in the near future (Table 3).

### Courses of pregnancies in women with testicular cancer patients as partners

Except for one child with a low birth weight (2225 g) (CT), all other 20 women had a normal course of pregnancy. Minor complications (bleeding and premature start of labour) were observed in two women. Nine of 21 pregnant women (43%) chose a higher rate of pregnancy check-up which was measured by the number of amniocenteses (four of 21), ultrasound examinations or physician consultations. Nineteen women had a spontaneous delivery, and in two women a Caesarian section was carried out. The mean birth weight and height were 3398.5 g (range 2225–4070) and 51.5 cm (range 46–56). Three spontanous abortions were reported in the total cohort. All abortions occurred before the 14th week of gravidity. Concerning the health status of the offspring, there were no major birth defects or complications during and after birth. All children have been reported to develop normally up to a median age of 62 months (range 1–180). Two children with congenital defects, one with cryptorchidism and one with hip dysplasia, were identified (Table 4).

### Sexual dysfunctioning

The incidences of various types of sexual dysfunctions are listed in Table 5. In total, 29 patients (30%) were found to have ejaculation problems after treatment. A significantly higher incidence was observed in patients who underwent RPLND alone (45%, $P$ = 0.03) or a secondary resection after chemotherapy (CT+SRRTM = 55%, $P$ = 0.01) as compared to patients treated with chemotherapy alone (11%). In three of 29 patients (10%) the

**Table 3** Fertility status in testicular cancer patients

| | | Treatment groups (No. of patients) | | | | | |
|---|---|---|---|---|---|---|---|
| | **All (patients)** | **SV** | **RT**[a] | **RPLND** | **CT** | **CT+SRRTU** | ***P*-value** |
| Fathered children before treatment | 39 (40%) | 0 | 4 (100%) | 8 (62%) | 14 (44%) | 13 (31%) | 0.03 |
| Fathered children after treatment | 21 (21%) | 2 (29%) | 2 (50%) | 2 (15%) | 7 (22%) | 8 (19%) | 0.90 |
| | | | | | 0.03 | | |
| Unfulfilled wish for children | 19 (19%) | 0 | 0 | 1 (7%) | 5 (16%) | 13 (31%) | 0.10 |
| Fertility testing | 15 (15%) | 0 | 0 | 1 (7%) | 5 (16%) | 9 (21%) | 0.42 |
| Plan to adopt children | 0 | 0 | 0 | 0 | 0 | 0 | – |
| Cryopreservation offered before treatment | 20 (20%) | 0 | 0 | 1 | 12 (38%) | 7 (17%) | 0.14 |
| Cryopreservation done | 8 (8%) | 0 | 0 | 0 | 3 (9%) | 5 (12%) | 0.66 |
| Plan in vitro fertilization | 2 (2%) | 0 | 0 | 0 | 1 (3%) | 1 (2%) | 1.0 |

[a] This subgroup of patients was not considered for statistical analysis comparing treatment groups. RPLND, retroperitoneal lymph node dissection; CT+SRRTU, chemotherapy and retroperitoneal lymph node resection (mostly lumpectomy); CT, chemotherapy; RT, radiotherapy; SV, surveillance; NE, not evaluable.

© Cancer Research Campaign 1999

**Table 4** Pregnancies in female partners of patients treated for testicular cancer

| | | Treatment groups (No. of patients) | | | | |
|---|---|---|---|---|---|---|
| | **All (= 21)** | **SV** | **RT** | **RPLND** | **CT** | **CT+SRRTU** |
| Increased prevention check up | 9 (43%) | 1 (33%) | 1 (50%) | 1 (50%) | 2 (29%) | 4 (50%) |
| Amniocentesis | 4 (19%) | 1 (33%) | 1 (50%) | – | – | 2 (25%) |
| Course of pregnancy | | | | | | |
| Normal | 19 (90%) | 1 (66%) | 2 (100%) | 2 (100%) | 7 (100%) | 7 (88%) |
| Complications | 2 (10%) | 1 (33%) | – | – | – | 1 (13%) |
| Miscarriage after treatment | 3 (3%) | – | 1 (25%) | – | 1 (3%) | 1 (2%) |
| Delivery | | | | | | |
| Spontanous | 16 (76%) | 2 (66%) | 2 (100%) | 2 (100%) | 5 (71%) | 5 (63%) |
| Caesarian section | 2 (10%) | – | – | – | 2 (29%) | – |
| Children | | | | | | |
| Mean birth weight | 3398.5 | 3000 (2400–3600) | 3700 (3700–3700) | 3500 (3500–3500) | 3425 (2225–4070) | 3431 (2950–3870) |
| Mean birth height | 51.5 (46–56) | 49.5 (46–53) | 53.0 (53–53) | 54.0 (52–56) | 51.2 (50–53) | 51.4 (49–56) |

RPLND, retroperitoneal lymph node dissection; CT+SRRTU, chemotherapy and retroperitoneal lymph node resection (mostly lumpectomy); CT, chemotherapy; RT, radiotherapy; SV, surveillance; NE, not evaluable.

**Table 5** Sexual dysfunction in men with testicular cancer

| | | Treatment groups (No. of patients) | | | | | |
|---|---|---|---|---|---|---|---|
| | **All (patients)** | **SV** | **RT**[a] | **RPLND** | **CT** | **CT+SRRTU** | ***P*-value** |
| | | | | 0.03 | 0.01 | | |
| Inability to ejaculate | 29 (30%) | – | – | 5 (45%) | 3 (9%) | 21 (55%) | <0.001 |
| Reduced semen volume | | | | | | | |
| Total | 16 (16%) | – | – | 4 (31%) | 5 (16%) | 7 (17%) | 0.38 |
| Slightly | 6 | – | – | 1 (8%) | 4 (13%) | 1 (2%) | 0.27 |
| | | | | 0.06 | | | |
| Severe | 10 | – | – | 3 (23%) | 1 (3%) | 6 (14%) | 0.12 |
| Dissatisfaction with sexual life | | | | | | | |
| Pretreatment | 8 (8%) | 1 (14%) | – | 1 (8%) | 2 (6%) | 7 (17%) | 0.60 |
| Post-treatment | 13 (13%) | 1 (14%) | – | 2 (15%) | 3 (9%) | 4 (10%) | 0.92 |
| Loss of sexual drive | | | | | | | |
| Pretreatment | 2 (2%) | – | – | – | 2 (6%) | – | 0.28 |
| Post-treatment | 7 (7%) | – | – | – | 1 (3%) | 6 (14%) | 0.18 |
| Reduced erectile potential | | | | | | | |
| Pretreatment | 1 (1%) | – | – | – | – | 1 (2%) | 1.0 |
| Post-treatment | 9 (9%) | – | – | – | 3 (9%) | 6 (14%) | 0.40 |

[a] This subgroup of patients was not considered for statistical analysis comparing treatment groups. RPLND, retroperitoneal lymph node dissection; CT+SRRTU, chemotherapy and retroperitoneal lymph node resection (mostly lumpectomy); CT, chemotherapy; RT, radiotherapy; SV, surveillance; NE, not evaluable.

ejaculatory function recovered between 1 and 3 years after treatment. Sixteen patients reported a decrease in the quantity of semen fluid production: six patients reported slight and ten patients severe reductions. The reduction of semen fluid quantity appears to be related to RPLND (31%, $P = 0.06$).

Eight per cent of patients reported dissatisfaction with their sexual life before the diagnosis of cancer. Two patients reported loss of sexual drive (2%) and one patient reported a reduced erectile potential (1%). A score to self-adjust the intensity of sexual life (including the frequency of sexual intercourse and the intensity of orgasms) showed no difference before and after treatment for all patients, with a mean score of 2.3 ($P = 0.51$). As expected, the score significantly decreased during treatment (data not shown), but recovered almost completely after treatment, with no differences between treatment groups. 'Loss of sexual drive', 'reduced erectile potential' and 'dissatisfaction with sexual life' were observed more frequently after treatment compared to pretreatment status but this was not statistically significant. The highest incidences regarding those disorders was seen in patients receiving CT+SRRTM (not significant). Age and duration of follow-up did not influence the incidence of sexual disorders.

### DISCUSSION

Despite a reasonable number of retrospective studies investigating the influence of treatment on sexual function, the question remains open whether and in what degree testicular cancer patients are at risk for sexual morbidity (Rieker et al, 1985, 1989; Schover and Eschenbach, 1985; Schover et al, 1986; Moynihan, 1987; Gritz et al, 1989; Stoter et al, 1989; Tinkler et al, 1992; Aass et al, 1993; Arai et al, 1997; Jonker-Pool et al, 1997). The available data vary widely and a correlation to different treatment modalities is lacking in most studies. Furthermore, the comparison of different studies is difficult due to differences in irradiation procedures,

 *© Cancer Research Campaign 1999*

chemotherapy regimens, composition of the cohort of patients and in the aims of each study. We report nearly 100 patients who underwent different treatment modalities for testicular germ cell tumour between 1970 and 1993. This descriptive study provides data about sexual function, fertility status/distress and the adaptive behavioural responses, and reports on the courses and outcome of pregnancies after treatment.

For testicular cancer survivors the highest child-bearing potential after treatment has been reported in seminoma patients who were treated with radiation therapy alone. The lowest rates were observed in patients who had undergone retroperitoneal surgery plus cisplatin-based chemotherapy (Rieker et al, 1985, 1989; Schover and Eschenbach, 1985; Petersen et al, 1994; Arai et al, 1997). In this series patients receiving CT+SRRTM reported childlessness more frequently compared to patients treated with CT alone. Despite the observation of a comparable decrease in subjective sexual aspects (libido, orgasm, satisfaction) in both treatment groups, the combined treatment resulted in a higher fertility distress. This is in accordance with a report by Arai et al (1997) who reported a 21% difference between both groups concerning desire for children (68% vs 47%). Compared to the frequency of unintentional childlessness in the German population (about 17%), only patients receiving both chemotherapy and retroperitoneal surgery appear to have a higher incidence of an unfulfilled wish for children, whereas for all other treatment groups the incidence was lower (Bruckert, 1991).

Today, sperm-banking may be beneficial even for patients who are subfertile at the time of diagnosis, because the techniques of sperm preservation and in-vitro fertilization rapidly advances. Sperm-banking awareness is one of several possible fertility adjustment responses, besides adoption awareness, fertility testing and trying to father children and fertility distress (Rieker et al, 1990). The psychological aspects of infertility, its psychosomatic components on health and sexuality have been described elsewhere (Burns, 1987). In the current investigation, 20 of 87 (23%) patients who underwent CT or RPLND reported that cryopreservation had been offered to them. Forty per cent of them had performed sperm-banking and two of those patients were planning in vitro fertilization. Although sperm-banking awareness was somewhat lower than described by Rieker et al (1990), the number of men who had performed sperm-banking was in the same range. In our investigation, none of the patients were planning to adopt children in the near future. In the above mentioned study, 21% of men considered adoption and 3% had adopted a child.

Although our cohort of patients with 21 born children is too small for definitive conclusions, we did not observe an elevated rate of complications during pregnancies in the partners of our testicular cancer patients. The rate of pregnancy check-up appeared to be higher as compared to the general population. Four of 21 women underwent amniocentesis (19%) and approximately 40% consulted their physician earlier than is the norm. Altogether, three of 26 pregnancies were miscarriages. No adverse pregnancy outcome defined as fetal or neonatal death or severe congenital malformation was observed, with the exception of the premature birth of a live-born infant weighing 2225 g. There are only limited data about the pregnancy outcome of partners of male cancer survivors (Brenner et al, 1985; Stoter et al, 1989; Senturia and Peckham, 1990; Byrne and Mulvihill, 1991). Another investigation regarding children fathered after the remaining gonad had been exposed to chemotherapy had also found no evidence of an increased risk of congenital malformations compared to matched controls (Dodds et al, 1993). A recent investigation showed no significantly increased risk of non-hereditary cancer among the offspring of survivors of cancer in childhood (Sankila et al, 1998). No pregnancies occurred during the actual cancer treatment period; patients were advised to perform birth control during therapy.

It has been demonstrated that testicular cancer can affect sexual function (Jonker-Pool et al, 1997). The incidences of subjective aspects, like ‘loss of libido’, ‘erectile dysfunction’ and ‘reduced satisfaction with sexual life’ were 7%, 9% and 13% respectively. Data available from the literature ranged from 4% up to 38% in different reports (Rieker et al, 1985; Schover and Eschenbach, 1985; Moynihan, 1987; Schover, 1987; Fossa et al, 1988; Gritz et al, 1989; Jonker-Pool et al, 1997). It has been suggested that sexual dysfunctions are related to the intensity and the modality of the treatment. A low incidence of sexual dysfunction was observed in surveillance patients or in seminoma patients undergoing irradiation (Rieker et al, 1985; Schover and Eschenbach, 1985; Jonker-Pool et al, 1997). Patients who underwent chemotherapy (± SRRTM) appeared to be at the highest risk for sexual dysfunction (Rieker et al, 1985; Arai et al, 1997; Jonker-Pool et al, 1997). With the exception of absence of ejaculation, no major difference has been reported between the CT and CT+SRRTM therapies (Arai et al, 1997; Jonker-Pool et al, 1997; van Basten et al, 1997*b*; own series). According to expectations, absence of antegrade ejaculation was reported by 45% and 55% ($P = 0.03$ and $P = 0.01$) of our patients undergoing RPLND or CT+SRRTM. Possible damage is related to the extent of the retroperitoneal surgical approach (Nijman et al, 1987; Jones et al, 1993). All of our patients with primary RPLND had stage II disease and underwent unilateral RPLND either as a radical procedure (before 1992) or as a modified RPLND (after 1992). Secondary resection of residual masses after chemotherapy was usually performed as a lumpectomy (Hartmann et al, 1997*a*, 1997*b*).

Absence of ejaculation was reported by three patients treated with CT alone (9%). Chemotherapy may affect the hormonal, vascular and nervous systems (van Basten et al, 1995, 1997*a*). All of these systems are important for sexual function and their disturbance may thus cause ejaculatory problems. This rare observation was reported previously by another investigator in 7% of chemotherapy-treated patients (Arai et al, 1997). A decreased erectile potential was observed in 9% of our patients, which is within the range of other investigations (Arai et al, 1997; Jonker-Pool et al, 1997). A higher incidence has been reported in patients undergoing radiation therapy (15–48%) (Arai et al, 1997; Jonker-Pool et al, 1997; van Basten et al, 1997*a*).

In conclusion, long-lasting sexual problems after therapy for testicular cancer are present in approximately one-fifth of patients undergoing treatment for testicular germ cell tumour. On the other hand, the majority of patients have not reported infertility or sexual dysfunction-related symptoms. Ejaculation disturbances were found in a large number of patients undergoing RPLND. The relative risk for infertility appears to be elevated for patients treated with the combination of CT+SRRTM leading to a definitve number of infertile men. The courses of pregnancies in partners of testicular cancer survivors did not differ from those of the general population and no major birth defects or serious chronic diseases were detectable in any of 21 children. This finding confirms other studies in long-term survivors of cancer treatment which revealed no increased risk for congenital abnormalities or late effects in the offspring.

© Cancer Research Campaign 1999

### ACKNOWLEDGEMENTS

The authors would like to thank the members of the Department of Gynaecology and the Department of Medical Psychology at Hanover University Medical School for their fruitful suggestions in the preparation of the questionnaire and Mrs Eva Bock for her help in preparing the manuscript.

### REFERENCES

Aass N, Fossa SD, Theodorson L and Norman N (1991) Prediction of long-term gonadal toxicity after standard treatment for testicular cancer. *Eur J Cancer* **27**: 1087–1091

Aass N, Grunfeld B, Kaalhus O and Fossa SD (1993) Pre- and posttreatment sexual life in testicular cancer patients: a descriptive investigation. *Br J Cancer* **67**: 1560–1567

Arai Y, Kawakita M, Okada Y and Yoshida O (1997) Sexuality and fertility in long-term survivors of testicular cancer. *J Clin Oncol* **15**: 1444–1448

Berthelsen JG and Skakkebaek NE (1984) Sperm counts and serum follicle-stimulating hormone levels before and after radiotherapy and chemotherapy in men with testicular germ cell cancer. *Fertil Steril* **41**: 281–286

Bloom JR, Fobair P, Gritz E, Wellisch D, Spiegel D, Varghese A and Hoppe R (1993) Psychosocial outcomes of cancer: a comparative analysis of Hodgkin's disease and testicular cancer. *J Clin Oncol* **11**: 979–988

Bokemeyer C (1998) Current trends in chemotherapy for metastatic nonseminomatous testicular germ cell tumors. *Oncology* **55**: 177–188

Bokemeyer C, Kohrmann O, Tischler J, Weissbach L, Rath U, Haupt A, Schöffski P, Harstrick A and Schmoll HJ (1996) A randomized trial of cisplatin, etoposide and bleomycin (PEB) versus carboplatin, etoposide and bleomycin (CEB) for patients with 'good-risk' metastatic non-seminomatous germ cell tumors. *Ann Oncol* **7**: 1015–1021

Brenner J, Vugrin D and Whitmore W (1985) Effect of treatment on fertility and sexual function in males with metastatic nonseminomatous germ cell tumours of tests. *Am J Clin Oncol* **8**: 178–182

Bruckert E (1991) How frequent in unintentional childlessness in Germany? *Andrologica* **23**: 245–250

Buena F, Swerdloff RS, Steiner BS, Lutchmansingh P, Peterson MA, Randian MR, Galmarini M and Bhasin S (1993) Sexual function does not change when serum testosterone levels are pharmacologically varied within the normal male range. *Fertil Steril* **59**: 1118–1123

Burns LH (1987) Infertility as boundary ambiguity: one theoretical perspective. *Fam Process* **26**: 359–372

Byrne J and Mulvihill JJ (1991) Long-term survivors of childhood and adolescent cancer: their fertility and the health of their offsprings. In *Complications of Cancer Management*, Plowman PN, McElwain TJ, Meadows AT (eds), pp. 114–127. Butterworth Scientific: Guildford

Cassileth BR and Steinfeld AD (1987) Psychological preparation of the patient and family. *Cancer* **60**: 547–552

Cavalli F, Monfadini S and Pizzocaro G (1980) Report on the international workshop on staging and treatment of testicular cancer. *Eur J Cancer* **16**: 1367–1372

Dodds L, Marrett LD, Tomkins DJ, Green B and Sherman G (1993) Case-control study of congenital anomalies in children of cancer patients. *Br Med J* **307**: 164–168

Drasga RE, Einhorn LH, Williams SD, Patel DN and Stevens EE (1983) Fertility after chemotherapy for testicular cancer. *J Clin Oncol* **3**: 179–183

Einhorn LH (1990) Treatment of testicular cancer: a new and improved model. *J Clin Oncol* **8**: 1777–1781

El-Beheiry A, Souka A, El-Kamshoushi A, Hussein S and El-Sabah K (1988) Hyperprolacteinia and impotence. *Arch Androl* **21**: 211–214

Fossa SD, Klepp O and Aakvaag A (1980) Serum hormone levels in patients with malignant testicular germ cell tumours without clinical and/or radiological signs of tumour. *Br J Urol* **52**: 151–157

Fossa SD, Ous S, Abyholm T, Norman N and Loeb M (1985) Post-treatment fertility in patients with testicular cancer. II. Influence of cis-platin-based combination chemotherapy and of retroperitoneal surgery on hormone and sperm cell production. *Br J Urol* **57**: 210–214

Fossa SD, Abyholm T, Norman N and Jetne V (1986) Post-treatment fertility in patients with testicular cancer. *Br J Urol* **58**: 315–319

Fossa SD, Aass N and Kaalhus O (1988) Testicular cancer in young Norwegians. *J Surg Oncol* **39**: 43–63

Goldstein I, Feldman MI, Deckers PJ, Babaian RK and Krane RJ (1984) Radiation-associated impotence. A clinical study of its mechanism. *JAMA* **251**: 903–910

Gritz ER, Wellisch DK, Wang HJ, Siau J, Landsverk JA and Cosgrove MD (1989) Long-term effects of testicular cancer on sexual functioning in married couples. *Cancer* **64**: 1560–1567

Guazzerri S, Lembo A, Ferro G, Artibani W, Merlo F, Zanchetta R and Pagano F (1985) Sperm antibodies and infertility in patients with testicular cancer. *Urology* **26**: 139–142

Harstrick A, Schmoll HJ, Kohne-Wompner CH, Bergmann L, Lammers U, Hohnloser J, Dolken G, Reichhardt P, Siegert W, Natt F, Rath V, Wilke H and Poliwoda H (1991) Cisplatin, etoposide, ifosfamide, vincristine and bleomycin combination chemotherapy for far advanced testicular carcinoma. *Ann Oncol* **2**: 197–202

Hartmann JT, Candelaria M, Kuczyk MA, Schmoll HJ and Bokemeyer C (1997*a*) Comparison of histological results from the resection of residual masses at different sites after chemotherapy for metastatic non-seminomatous germ cell tumours (NSGCT). *Eur J Cancer* **33**: 843–847

Hartmann JT, Schmoll HJ, Kuczyk MA, Candelaria M and Bokemeyer C (1997*b*) Post-chemotherapy resections of residual tumor masses from metastastic non-seminomatous germ cell tumours. *Ann Oncol* **8**: 531–538

Hendry WF, Stedronska J, Jones CR, Blackmore CA, Barrett A and Peckham MJ (1983) Semen analysis in testicular cancer and Hodgkin's disease: pre- and post-treatment findings and implications for cryopreservation. *Br J Urol* **55**: 769–773

Jones DR, Norman AR, Horwich A and Hendry WF (1993) Ejaculatory dysfunction after retroperitoneal lymphadenectomy. *Eur Urol* **23**: 169–171

Jonker-Pool G, van Basten JP, Hoekstra HJ, van Driel MF, Sleijfer DT, Koops HS and van de Wiel HB (1997) Sexual functioning after treatment for testicular cancer: comparison of treatment modalities. *Cancer* **80**: 454–464

Lampe H, Horwich A, Norman A, Nicholls J and Dearnaley DP (1997) Fertility after chemotherapy for testicular germ cell cancers. *J Clin Oncol* **15**: 239–245

Loehrer PJ Sr, Lauer R, Roth BJ, Williams SD, Kalasinski LA and Einhorn LH (1988) Salvage therapy in recurrent germ cell cancer: ifosfamide and cisplatin plus either vinblastine or etoposide. *Ann Intern Med* **109**: 540–546

Moynihan C (1987) Testicular cancer: the psychosocial problems of patients and their relatives. *Cancer Surv* **6**: 477–510

Nijman JM, Schraffordt-Koops HS, Kremer J and Sleijfer DT (1987) Gonadal function after surgery and chemotherapy in men with stage II and III non-seminomatous testicular tumors. *J Clin Oncol* **5**: 651–656

Petersen PM, Hansen SW, Giwercman A, Rorth M and Skakkebaek NE (1994) Dose-dependent impairment of testicular function in patients treated with cisplatin-based chemotherapy for germ cell cancer. *Ann Oncol* **5**: 355–358

Rieker PP, Edbril SD and Garnick MB (1985) Curative testis cancer therapy: psychosocial sequelae. *J Clin Oncol* **3**: 1117–1126

Rieker PP, Fitzgerald EM, Kalish LA, Richie JP, Lederman GS, Edbril SD and Garnick MB (1989) Psychosocial factors, curative therapies, and behavioral outcomes. A comparison of testis cancer survivors and a control group of healthy men. *Cancer* **64**: 2399–2407

Rieker PP, Fitzgerald EM and Kalish LA (1990) Adaptive behavioral responses to potential infertility among survivors of testis cancer. *J Clin Oncol* **8**: 347–355

Sankila R, Olsen JH, Anderson H, Garwicz S, Glattre E, Hertz H, Langmark F, Lanning M, Moller T and Tulinius H (1998) Risk of cancer among offspring of childhood-cancer survivors. *N Engl J Med* **338**: 1339–1344

Schmoll HJ, Harstrick A, Bokemeyer C, Dieckmann KP, Clemm C, Berdel WE, Souchon R, Schöber C, Wilke H and Poliwoda H (1993) Single-agent carboplatinum for advanced seminoma. A phase II study. *Cancer* **72**: 237–243

Schover LR (1987) Sexuality and fertility in urologic cancer patients. *Cancer* **60**: 553–558

Schover LR and von Eschenbach AC (1985) Sexual and marital relationships after treatment for non-seminomatous testicular cancer. *Urology* **25**: 251–255

Schover LR, Gonzales M and von Eschenbach AC (1986) Sexual and marital relationships after radiotherapy for seminoma. *Urology* **27**: 117–123

Senturia YD and Peckham CS (1990) Children fathered by men treated with chemotherapy for testicular cancer. *Eur J Cancer* **4**: 429–432

Sleijfer S, van der Mark TW, Schraffordt-Koops H and Mulder NH (1995) Decrease in pulmonary function during bleomycin-containing combination chemotherapy for testicular cancer: not only a bleomycin effect. *Br J Cancer* **71**: 120–123

Stoter G, Koopman A, Vendrik CP, Struyvenberg A, Sleyfer DT, Willemse PH, Schraffordt Koops H, van Oosterom AT, ten Bokkel Huinink WW and Pinedo HM (1989) Ten-year survival and late sequelae in testicular cancer patients treated with cisplatin, vinblastine, and bleomycin. *J Clin Oncol* **7**: 1099–1104

Tinkler SD, Howard GC and Kerr GR (1992) Sexual morbidity following radiotherapy for germ cell tumours of the testis. *Radiother Oncol* **25**: 207–212

 *© Cancer Research Campaign 1999*

Tomic R, Bergman B, Damber JE, Littbrand B and Löfroth PO (1983) Effects of external radiation therapy for cancer of the prostate on the serum concentrations of testosterone, luteinizing hormone and prolactine. *J Urol* **130**: 287–290

van Basten JP, Jonker Pool G, van Driel MF, Sleijfer DT, van der Wiel HB and Hoekstra HJ (1995) The sexual sequelae of testicular cancer. *Cancer Treat Rev* **21**: 479–495

van Basten JP, Hoekstra HJ, van Driel MF, Koops HS, Droste JH, Jonker Pool G, van de Wiel HB and Sleijfer DT (1997*a*) Sexual dysfunction in nonseminoma testicular cancer patients is related to chemotherapy-induced angiopathy. *J Clin Oncol* **15**: 2442–2448

van Basten JP, Jonker-Pool G, van Driel MF, Sleijfer DT, Droste JHJ, van de Wiel HBM, Koops HS, Molenaar WM and Hoekstra HJ (1997*b*) Sexual functioning after multimodality treatment for disseminated nonseminomatous testicular germ cell tumor. *J Urol* **158**: 1411–1416

von Eschenbach AC (1980) Sexual dysfunction following therapy for cancer of the prostate, testis and penis. *Front Radiat Ther Oncol* **101**: 109–116

Williams SD, Birch R, Einhorn LH, Irwin L, Greco FA and Loehrer PJ (1987) Treatment of disseminated germ-cell tumors with cisplatin, bleomycin, and either vinblastine or etoposide. *N Engl J Med* **316**: 1435–1440

*© Cancer Research Campaign 1999*
